# Periodontal Disease and Its Association with *Porphyromonas gingivalis*: Current Understanding of Microbial Dysbiosis, Immunopathology and Immune Evasion

**DOI:** 10.3390/microorganisms14030641

**Published:** 2026-03-12

**Authors:** Samantha Robins, Alex Strachan, Vehid Salih, Andrew Foey

**Affiliations:** 1 School of Biomedical Sciences, University of Plymouth, Plymouth PL4 8AA, UK; samantha.robins@plymouth.ac.uk; 2 Plymouth Electron Microscopy Centre, University of Plymouth, Plymouth PL6 8BT, UK; alexander.strachan@plymouth.ac.uk; 3 Peninsula Dental School, University of Plymouth, Plymouth PL4 8AA, UK; vehid.salih@plymouth.ac.uk

**Keywords:** periodontal disease, *Porphyromonas gingivalis*, dysbiosis, inflammation, immune evasion, endotoxin tolerization, inflammation

## Abstract

*Porphyromonas gingivalis* is described as a keystone pathogen associated with periodontal disease (PD), which exhibits enhanced representation upon microbial dysbiosis in such a chronic inflammatory disease. This oral pathogen drives and contributes to a dysregulated immune response, resulting in stages of aggressive destructive immune activation and inflammation punctuated by immune suppression, which underlies the relapsing–remitting nature of this disease. The understanding of key mechanisms and balance between protective innate, adaptive immune responses and dysregulated responses, linked to changes in the oral mucosal microbial environment, will afford researchers the potential to manipulate oral mucosal environments for clinical benefit. This review focuses on the dynamic interactions between the oral pathogen *P. gingivalis* and the immune system with an emphasis on immune evasion and how the potential correction of these mechanisms may benefit future therapeutic interventions, leading to the successful treatment of PD.

## 1. Introduction to Periodontal Disease

The human oral cavity accommodates approximately 700 species of bacteria. Together, these communities comprise the oral microbiome [1], a rich and diverse blend of microorganisms adhering to the surfaces of teeth, as well as one another, and located within the gingival sulcus as biofilms. Any change, or dysbiosis, in this community and interaction of the bacteria between themselves and/or the oral tissues can lead to oral and, specifically, periodontal disease (PD). PD has been estimated to affect up to 50% of the global population, and it is considered one of the most prevalent chronic conditions with over 1 billion people impacted by the disease [2]. Affecting mainly adults, it is one of the most prominent diseases, particularly among older adults [3]. PDs comprise a range of inflammatory conditions affecting the supporting structures of the teeth, i.e., the gingiva, periodontal ligament and alveolar bone, and include gingivitis (reversible) and periodontitis (non-reversible). Gingivitis is the reversible stage of disease as a result of excessive plaque and features erythematous bleeding and swollen gingiva, which can be managed effectively with improved oral hygiene care. However, PD is irreversible, due to the painful infection and permanent destruction of underlying periodontal tissues, especially of the periodontal ligament attachments to the alveolar bone, as well as gingival tissue, and often requires surgical intervention. PD causes gingiva to recede, exposing the collagenous tissues that connect the soft tissue (gingiva, periodontal ligament) to the hard tissues of the cementum and bone, creating deeper pockets in the gingival sulcus and allowing microbial products and enzymes to erode the healthy bone beneath. This progressive destruction of both soft and hard tissues is meditated by a complex array of interchange between the dysbiosis of the microbial community and the aberrant immune responses within the tissues [4]. In 2017, a periodontal classification jointly defined by the American Association of Periodontology and European Federation of Periodontology defined periodontitis as a chronic and multifactorial inflammatory disease leading to clinical attachment loss, the pocketing of the sulcus and gingival bleeding as characteristic signs. The new classification describes the new multi-dimensional severity and complexity of the disease and how to grade it [5]. PD can be graded depending on the rate of progression, responsiveness to standard therapy and its impact on systemic health. The clinical attachment loss (CAL) of tissues may be considered as slow, moderate or rapid after 5 years of diagnosis.

Putative periodontal pathogens are enhanced in dysbiosis, yet good oral hygiene and care, alongside the therapeutic removal of the advanced biofilm (plaque), can ameliorate inflammation. It is important to acknowledge that the severity of the disease depends on environmental and host risk factors, both modifiable (e.g., smoking) and non-modifiable (e.g., genetic susceptibility). Continued tissue destruction induces a continuous positive feedback loop of proteolysis, inflammation, and beneficial conditions for periodontal pathogens. Keystone microbial pathogens and sustained gingival inflammation are critical to periodontal disease progression. One such keystone bacterium has been identified, and considerable research has shown that *Porphyromonas gingivalis* (*P. gingivalis*), a Gram-negative anaerobic bacterium, is the major etiologic agent which contributes to chronic periodontitis [6]. Furthermore, the impact of chronic inflammatory diseases at sites distal to the oral cavity on periodontitis, and the defined role of periodontitis in systemic inflammation, is also becoming recognized in PD pathogenesis [7].

## 2. The Oral Microbiota in Health and Periodontal Disease

The oral microbiota plays a prominent role in dictating the health of the oral mucosa and teeth, effectively controlling immune activation or tolerance. The healthy oral microbiota is predominated by bacterial populations consisting of Gram-positive aerobic bacteria such as *Streptococcus sanguinis*, *Streptococcus oralis*, *Actinomyces naesludii* and specific genera including *Veillonella* spp., *Neisseria*, *Rothia*, *Corynebacterium* and *Actinomyces*, with an appreciable scarcity of spirochetes and motile bacilli [8]. With inflammatory progression to gingivitis, the microbiota changes, with up to half the population being represented by Gram-positive cocci and a slight increase in spirochetes and motile bacteria, with an accumulation of *Prevotella intermedia*, *Veillonela* spp. and the scaffold bacterium *Fusobacteria nucleatum*, to which these later colonizing pathogenic bacteria adhere. The progression of early gingivitis to longer-term destructive periodontitis results in the dysbiosis of the oral microbiome in favour of Gram-negative obligate anaerobes predominated by motile rods and spirochetes, where the genera *Eubacterium*, *Campylobacter*, *Treponema* and *Tannerella* are associated with chronic PD and a strong correlation between dysbiosis and pocket depth with *Porphyromonas*, *Treponema*, and *Tannarella* over *Rothia* and *Corynebacterium* [8]. This dysbiotic periodontitis-associated microbiome contains orange complex bacteria *F. nucleatum* and *Prevotella intermedia* [9]; the green complex *Aggregatibacter actinomycemcomitans*; and red complex bacteria *Tanerella forsythia*, *Treponema denticola* and *Porphyromonas gingivalis* [10,11,12,13]. In addition, acute periodontitis is associated with increased colonization by both *A. actinomycemcomitans* and *P. gingivalis* [14,15], whereas the red complex bacteria are strongly associated with advanced periodontitis. Such changes in microbial populations and their aggregation in biofilms, such as plaque, will impact the availability and recognition of the microbial virulence factors utilized in the activation, suppression or deviation of host–anti-pathogen immune responses determining the inflammatory pathogenic mechanisms associated with PD.

## 3. *Porphyromonas gingivalis* Virulence Factors

With the role of *P. gingivalis* as the keystone pathogen driving periodontitis, the virulence factors produced by this pathogen play a vital role in bacterial growth and the evasion and modulation of host–anti-pathogen responses, hence adaptation to hostile host–anti-pathogen environments, such as oxidative stress created by respiratory burst activity. Integral to the survival and pathogenesis of this bacterium are the virulence factors fimbriae, LPS, haemolysin, haemagglutinin, gingipains and outer membrane vesicles (OMVs). Fimbriae mediate binding to host cells and the co-aggregation of plaque-forming bacterial species *A. naeslundii*, *S. gordonii* and *S. oralis*. *P. gingivalis* invades gingival epithelial cells [16], where phagocytosis by these oral epithelial cells induces cell autophagy, enabling bacterial replication, hence persistence, whilst suppressing apoptosis [17]. Fimbria protein subunits exist as long and short forms, namely FimA and Mfa1, respectively [12,13,18,19]. Short fimbriae bind the SspA and SspB proteins of *S. gordonii*, hence further facilitating bacterial aggregation and biofilm formation [12,20]. Relevant to PD, these short fimbriae also induce osteoclast (OC) differentiation and enhance bone resorption via the production of IL-1β, TNFα, IL-6 and RANKL [12,21]. Long FimA subunits, on the other hand, bind host tissues and epithelial cells via interaction with glycoproteins, fibrinogen, fibronectin, hydroxyapatite and lactoferrin [12,22,23]. In addition, long fimbriae are also capable of facilitating resistance to the innate immune complement system [12] and binding TLR2, hence initiating an inflammatory response mediated via the production and secretion of a plethora of cytokines (including IL-1β, IL-6, IL-8, TNFα) (Section 4), as well as the selective impairment of IL-12 production, with a consequential suppression of Th1 activation and cell-mediated immunity (CMI) impacting bacterial clearance [24]. The mechanism by which long fimbriae, FimA, impair IL-12 is dependent on their binding partner; they can bind the complement receptor, CR3, which inhibits TLR-2-induced IL-12 via an ERK MAPK-dependent system [25,26]. Additionally, binding CXCR4 can cross-regulate TLR-2-mediated signalling by activating a cAMP-dependent PKA response which serves to inhibit NF-kB activation [27], hence suppressing NF-kB-dependent cytokines such as IL-12 and also IL-1β, TNFα, and IL-6.

The cysteine protease gingipains Rgp and Kgp produced by *P. gingivalis* are capable of degrading the extracellular matrix (e.g., collagen), cytokines, immunoglobulins and complement components [28], participating in bacterial co-aggregation, biofilm formation, the suppression of clotting, capillary permeability and the increased bleeding of periodontal tissues. Gingipains can also regulate host immunity by the degradation of the antimicrobial peptides (AMPs) α- and β-defensins; the down-regulation of macrophage CD14 expression; and the digestion of C3, C4 and C5 complement [29], hence suppressing inflammation whilst reducing bacterial elimination.

Critical to the survival of *P. gingivalis* is the supply of iron. The virulence factors haemolysin and haemagglutinin help liberate a supply of haem, which is a vital source of iron, for the survival and replication, and hence virulence, of this pathogen. Haem can be released from haemoglobin by the proteolytic activity of another key virulence factor, the gingipain Kgp [30,31,32,33]; hence *P. gingivalis* takes advantage of inflammatory bleeding by utilizing the virulence factors fimbriae, haemagglutinin, haemolysin and gingipains to agglutinate and haemolyze red blood cells, key to this liberation of iron. Finally, the availability of iron–haem directly affects the structure of LPS, hence its potency of inducing an inflammatory reaction by the innate immune system.

Both iron availability and gingipain functionality overlap at the level of expression, structure and functional potency of the Gram-negative bacterial pathogen-associated molecular pattern (PAMP) Lipopolysaccharide (LPS). As a consequence of iron availability, LPS can exist in distinct structural isoforms which are determined by their level of acylation (acyl chains of lipid A fatty acids) or degree of phosphorylation. In fact, haemin availability has been suggested to trigger the switch in *P. gingivalis* lipid A structure [34,35], and these lipid A modifications may influence haem availability [36]. Low haem levels result in the expression of the strong immunogenic penta-acylated LPS-1690, whereas higher concentrations induce the weaker immunogenic tetra-acylated LPS 1435/1449 form [37]. *P. gingivalis* LPS (PG-LPS) is known to signal through TLR4 and is associated with periodontal bone loss [12,38,39]; however, the LPS isoform determines the inflammatory response whereby, based on their proteomic mass:charge ratio, LPS1690 activates inflammatory pathways via NF-kB and p38 MAPK and reduces the intracellular survival of *P. gingivalis*, through its ability to induce human β-defensins-1, -2 and -3, whereas LPS1435/1449 is known to avoid the inflammatory response and possibly acts as a competitive inhibitor to TLR4-mediated inflammation and suppresses AMP production [40], as a protective evasion mechanism.

*P. gingivalis* also expresses a secreted peptidyl arginine deiminase (PPAD) enzyme, a virulence factor that modifies proteins by the process of the deimination of arginine, which converts it to citrulline [41,42,43]. This citrullination modifies the host protein structure, which can activate the immune system by exposure to DAMPs or by altered self-inducing autoimmunity [44,45], with this break in self-tolerance resulting in the production of ACPAs in PD [46]. In fact, the resulting citrullinated peptides of fibrinogen and a-enolase have been described as major auto-antigens associated with RA [44]. The effects of the PPAD citrullination of host proteins further benefits the virulence and survival of *P. gingivalis* by modifying innate defences and barrier functionality, where citrullinated C5a exhibits a loss of function which impacts its ability to initiate inflammatory mechanisms [47], and citrullinated EGF results in breaking epithelial–periodontal tissue barriers [43]. As such, PPAD and other virulence factors play a significant role in the immunopathogenesis of *P. gingivalis* infection in PD.

## 4. PD Immunopathology and Immune Response to *P. gingivalis*

PD inflammation progresses from a local response to a chronic inflammatory lesion and is associated with the dysbiosis of the oral microbiome [48,49,50]. The combined contribution of bacterial co-infection, biofilm formation and localized tissue-specific environmental control of virulence factors has a profound effect on the modulation of host immune responses to *P. gingivalis*, and these subverted responses characterize the relapsing–remitting nature of this chronic inflammatory pathology with an ever-changing effect on the oral microbiome and virulence of this pathogen. Inflammation increases the availability of haem, iron and metabolic products which favour a hypoxic/anaerobic environment with knock-on effects altering the proportion of Gram-negative proteolytic red complex bacteria [51,52,53,54]. As such, this hinders the understanding of whether dysbiosis triggers the inflammatory response or is a consequence of acute inflammation, contributing to the perpetuation of a chronic inflammatory response. Thus, the bi-directional crosstalk between the oral microbiome and host immune cells dictates this changing in inflammatory response to benefit either the host or pathogen. During the progression of PD, host immune responses are characterized by dysregulated innate inflammatory responses with knock-on effects and changes between cell-mediated immunity, humoral responses and the immune regulation/suppression of adaptive immunity that contribute at different stages of PD to characterize the relapsing/remitting nature of this pathology.

Within days of plaque accumulation, the bacterial PAMPs peptidoglycan (PGN) and lipoteichoic acid (LTA) of early colonizing Gram-positive aerobic bacteria activate complement, inducing the anaphylatoxins C3a and C5a, which trigger mast cell activation and the consequent vascular permeability and oedematous inflammation [55]. Integral to the triggering and initiation of acute inflammation, gingivitis and its progression towards periodontitis make up the dysregulated response of innate immune cells such as neutrophils (Nϕs) and macrophages (Mϕs) to *P. gingivalis*-derived PAMPs and virulence factors. Dependent on the biofilm environment and accessibility to haem, PG-LPS can be synthesized in the two isoforms mentioned above, with distinct consequences for PAMP recognition and inflammatory responsiveness. In general, *P. gingivalis*-derived PAMPs, such as fimbriae and LPS, activate tissue-resident epithelial cells and immune cells (Mϕs, Nϕs, mast cells and DCs); this results in these innate cells secreting a plethora of immune-modulatory cytokines that exhibit both pro-inflammatory (IL-1α, IL-1β, IL-6, IL-8, IL-12, IL-18) and anti-inflammatory (IL-6, IL-10, IL-11, IL-35) behaviour [Table 1]. Chemokine expression (IL-8, MCP-1, MIP-1α) induces an influx of Nϕs and monocytes which serve to amplify this inflammatory response and drive chronic inflammation. This persistence is facilitated by infiltrating Nϕs, through enhanced chemokine expression, arachidonate metabolites, proteolytic enzymes (matrix metalloproteinases, MMPs), NET (neutrophil extracellular trap) formation and the release of reactive oxygen species (ROS) [56]. In addition, Mϕ inflammasome activation results in enhanced maturation and secretion of IL-1β/IL-18, as well as inducing pyroptosis, which is an inflammatory cell death characterized by the release of danger-associated molecular patterns (DAMPs) that perpetuate inflammatory initiation through PRR signalling.

The immunopathology of established gingival lesion and chronic inflammatory responses is determined by this initial acute response and the persistence of virulence factors, PAMPs, DAMPs and the resulting immune cells recruited and activated in the oral mucosae. This immune–oral microbiome axis is integral to determining the ensuing adaptive immune responses elicited in this mucosal tissue, which is dependent on the cytokine profiles elicited in response to such pathogens as *P. gingivalis*. The cytokines produced and detected in saliva, GCF and oral mucosal tissue are summarized in Table 1, and they drive the recruitment and activation of innate immune cells (Nϕs, Mϕs, DCs, mast cells) and adaptive immune cells (Th1, Th2, Th17, Tr and B cells), with a shift away from Nϕ involvement and a consequent prevalence of Mϕ and lymphocyte (both T- and B-cell) involvement [119,120].

Th1 may be considered bacterial-resolving and secrete IFNγ and TNFα upon differentiation by IL-12; these cells effectively activate and differentiate pro-inflammatory M1 macrophage subsets, resulting in a tissue-destructive DTH (delayed-type hypersensitivity) response and Nϕ NET formation. PG-LPS has been described to influence Th2 differentiation and activation which, in an IL-4-rich environment, drives Mϕ polarization to an M2 subset, as well as B-cell activation and antibody secretion by plasma cells. Such subversion of Th1 to Th2 effectively changes the *P. gingivalis*-clearing CMI response to a less efficient humoral response, facilitating the persistence of *P. gingivalis* infection [121,122,123] and disease progression [124]. Historically, PD and its development and prognosis were considered to be determined by a Th1/Th2 paradigm; this has since been overtaken by the realization of a plastic inter-relation between Th17 and Treg subsets, where an intermediary phenotype of IL-17^+^/FoxP3^+^ cells (Treg to Th17 conversion) has been suggested to be present in periodontitis lesions [125]. Finally, with the persistence of *P. gingivalis*, the adaptive response progresses, in the presence of IL-6 and IL-23, to a Th17-driven mechanism [126]. Th17 cells are expanded in PD and secrete GM-CSF, TNFα, IL-21, IL-22 and IL-17, which potently activate Mϕs and Nϕs, perpetuating a cycle of tissue-destructive inflammation [127]. In fact, one of the end-stage factors associated with PD is the resorption and loss of alveolar bone, subsequently resulting in tooth loss. With the collaboration of both innate and adaptive cells, pro-inflammatory cytokines such as IL-1β and TNFα have been described to up-regulate RANKL, which can be secreted or expressed on the surface of Th1, Th17, B cells, osteoblasts (OBs) and natural killer T cells (NKTs), activating osteoclast (OC)-mediated bone resorption upon RANK ligation. Such RANKL-RANK-mediated OC bone resorption activity can be antagonized by OPG, a decoy receptor effectively inhibiting RANKL-RANK reception; future research may investigate the manipulation of such an OPG:RANKL ratio to control alveolar bone loss in PD [128].

The involvement of Th1, Th17 and Th2 cells may indicate the range of pro-inflammatory mechanisms involved in PD but fail to fully explain the episodes of remission where inflammatory destruction is effectively quiescent or actively suppressed. There are potentially several pathways by which these inflammatory mechanisms are tolerized or subverted: these include (i) negative regulation of TLR-induced pro-inflammatory Mϕs or endotoxin tolerization (ET); (ii) Mϕ subset plasticity—changing the M1 Mϕ subset to an anti-inflammatory M2 subset; and (iii) an Mϕ/APC cytokine profile exhibiting a TGFβ/IL-10 predominance with consequential differentiation favouring Tregs. Such immune-suppressive or immune deviation mechanisms are integral to the evasion strategies harnessed by *P. gingivalis* to ensure its persistence and may represent future therapeutic targets to control PD.

## 5. Immune Evasion

Rather than simply triggering inflammation, *P. gingivalis* is skilled with evasion mechanisms to modulate both host innate and adaptive immunity [129], allowing it to persist and promote a dysbiotic biofilm and continue in its destructive path [130]. Early innate defenders, including Nϕs and Mϕs, are undermined through subversive signalling [131]. Central to this activity are the arginine- and lysine-specific cysteine proteases, gingipains. Gingipains have been demonstrated to alter Nϕ function by increasing ROS, reducing Nϕ elastase (a serine protease required for microbial killing), and suppressing the expression of the Nϕ chemotactic cytokine IL-8 [132]. These changes suggest that gingipains diminish Nϕ recruitment and elastase-mediated killing, thereby facilitating *P. gingivalis* immune evasion, hence the persistence of this keystone pathogen. One study also showed that *P. gingivalis* prolongs Nϕ survival by up-regulating antiapoptotic BCL-2 family proteins. Although this effect was not directly gingipain-dependent, it likely contributes to the chronic inflammatory environment in which *P. gingivalis* thrives. These apparent contradictions in Nϕ involvement; diminished recruitment, yet increased survival, increased ROS production but reduced elastase, may be explained by both stage of PD progression and the evasion strategies employed by this bacterium. This probably reflects the early recruitment, activation and antimicrobial killing activity of Nϕs, whereas with the progression of PD and deployment of evasion strategies, *P. gingivalis* modulates Nϕ-mediated immunity in its favour, reducing recruitment, increasing the survival of killing-incompetent cells, and diminishing the levels of elastase yet being able to maintain a bacterial-supportive inflammatory environment.

Gingipains have similarly been connected to the suppression of dendritic cell activation [133]. However, the *P. gingivalis* strain W50 lacks this suppressive effect due to a HagA point mutation that disrupts the PorSS/gingipain secretion system, highlighting strain-specific differences in gingipain-mediated immune evasion. Gingipains additionally contribute to vascular dysfunction, where *P. gingivalis* degrades platelet endothelial cell adhesion molecule-1 (PECAM-1 or CD31), compromising endothelial integrity; increasing permeability, hence vascular leakage; and enabling the translocation of other bacteria [134]. Although infection enhances monocyte adhesion, gingipains impair their trans-endothelial migration by down-regulating CD99 and CD99L2 on endothelial cells and monocytes, indicating the disruption of normal immune cell trafficking.

*P. gingivalis* also thrives by uncoupling TLR2-driven inflammation from effective bacterial clearance. *P. gingivalis* also exploits the integrin-associated protein CD47, a ‘don’t eat me’ signal associated with some cancers, that suppresses phagocytic clearance via its interaction with the negative regulatory receptor SIRP-1a [135]. CD47 interacts with TLR2 to promote cell survival, whereas CD47 deficiency enhances Mϕ bacterial clearance. *P. gingivalis* further induces the secretion of thrombospondin-1 (TSP-1), a CD47 ligand that suppresses Nϕ bactericidal activity in relation to PD-associated bacteria. TLR2–CD47 co-signalling and TSP-1 induction suggest a major immune evasion pathway supporting *P. gingivalis* persistence. In fact, TSP-1 is elevated in the gingival crevicular fluid (GCF) of patients with PD and in inflamed gingiva in animal models, with PG-LPS driving its production [136]. In periodontal fibroblasts, TSP-1 promotes the expression of MMP-2 and MMP-9 and an increased RANKL/OPG ratio, all contributing to extracellular matrix degradation and bone resorption, while its inhibition suppresses cytokine production in PG-LPS-stimulated Mϕs. By amplifying inflammation and tissue destruction without improving bacterial clearance, TSP-1 supports a dysbiotic environment favourable to *P. gingivalis*.

*P. gingivalis* can establish the evasion of host defence mechanisms via a combination of immune suppression and immune deviation. The innate immune system can be deviated or suppressed by multiple simulation of PRRs through endotoxin tolerization (ET). Which mechanism predominates is likely dictated by the local environment determined by the stage of dysbiosis and immune response. *P. gingivalis* evades host defences through modifications of its LPS, including tetra-acylated and monophosphorylated lipid A forms [137,138], which act as weak TLR4 agonists, resulting in a reduced TLR-4-mediated induction of AMPs involved in epithelial defences and reduced inflammasome activation [139]. PG-LPS weakly activates the M1 and M2 Mϕ expression of pro-inflammatory cytokines and costimulatory molecules, whereas it elevates Arginase I expression in M2, in a PG-LPS isoform-dependent manner [140]. Such LPS-induced effects are capable of reducing the pro-inflammatory responses of M1 Mϕs while increasing M2 polarization, hence biasing towards an anti-inflammatory response and facilitating bacterial persistence, through a process of immune deviation. *P. gingivalis*-OMV gingipains mediate Mϕ CD14 loss, hence a reduction in TLR4-LPS responsiveness [141]. In contrast to this suppressive mechanism associated with OMV gingipains, *P. gingivalis* OMVs are enriched in C4′-monophosphoryl lipid A (C4′-MPLA), a potent TLR4 agonist that stimulates IFNβ and IL-1β production while diverting immune recognition away from the bacterium itself, suggestive of immune deviation rather than suppression [142]. Strains producing more OMVs (e.g., PG381) generated stronger pro-inflammatory responses. AMPs such as Cathelicidin (LL-37) blocked OMV-induced TLR4 signalling, confirming the immunostimulatory role of C4′-MPLA; however, PG NPLA-containing OMVs resist LL-37-mediated killing. Finally, studies focused on ET have demonstrated Mϕ subsets to exhibit differential sensitivity to ET induced by PG-LPS, where M2 Mϕs (anti-inflammatory subset) are selectively tolerized, and M1 Mϕs (pro-inflammatory subset) are refractory to tolerance induction, with respect to the induction of TNFα [143], whereas ET induced by repeated stimulation with *E. coli* K-12 LPS resulted in a uniform suppression of TNFα between both Mϕ subsets yet differing effects on IL-6 and IL-10 in a manner associated with the differential subset-specific expression of the negative regulators IRAK-M and Tollip [144].

In addition to the above, other established immune evasion strategies of *P. gingivalis* include immune deviation to adaptive immunity, with respect to T-cell responses, where Th17 is favoured over Treg immunity, due to the gingipain inhibition of IL-2- and IL-12-dependent Th1 differentiation [145]. T cells can also be suppressed, where PG-LPS was found to induce IDO expression in human gingival fibroblasts, which resulted in the indirect tolerization of T cells [146]. Recent evidence shows that *P. gingivalis* also manipulates host gene regulation to evade immunity. An RNA-seq analysis of wildtype- and gingipain-deficient *P. gingivalis*-stimulated Mϕs revealed widespread changes in alternative splicing, including the increased expression of the negative-regulatory PD-L1 and a preferential shift towards a PD-L1 isoform with higher PD-1 binding affinity, thus impairing and tolerizing adaptive immune T-cell function [147].

## 6. Current Treatments for Periodontitis

Depending on the advancement of the disease, less invasive procedures include scaling, root planing (smoothing of root surfaces to minimize bacterial attachment) and courses of antibiotics [148]. However, if PD is advanced, then more invasive treatments are required, and these increase in terms of severity, including pocket reduction surgery, soft tissue grafts, bone grafts, and guided tissue regeneration using both natural and synthetic biomaterials [149,150]. More recently, light-activated compounds have been utilized as a non-surgical treatment in the form of antimicrobial photodynamic therapy (aPDT) [151], and a variety of growth factors, e.g., Emdogain (containing enamel matrix proteins to help regenerate lost tissue) have also been applied [152]. As such, the current therapeutic approaches are focused on tissue replacement, regeneration and antimicrobial targeting; there is a huge gap between our understanding of the cellular and molecular immunopathological mechanisms driving PD and the translation to adopt potential immune-modulatory therapies.

## 7. Summary and Future Perspectives

**Figure 1:**
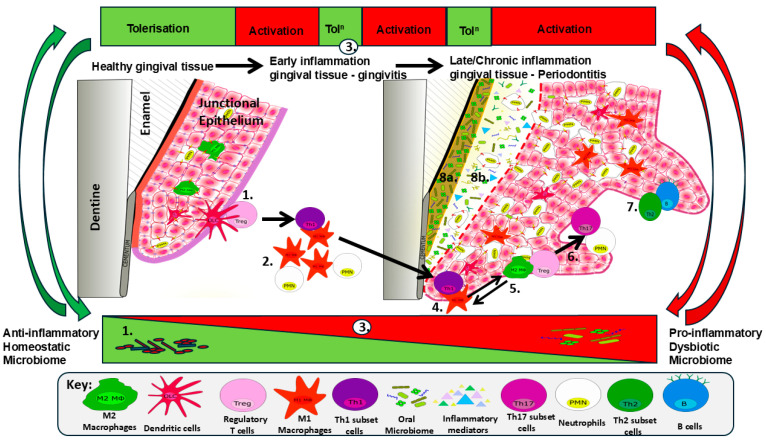
**The inter-relationship of *P. gingivalis*, microbiome, immunity and immune evasion determines PD.** The balance between healthy gingival and periodontal tissue homeostasis of the oral microbiome and dysbiosis associated with PD progression and chronicity is represented by the predominance of Gram-positive cocci in the tolerogenic/anti-inflammatory environment in the green triangle at the bottom of the figure and its progression to Gram-negative rods and spirochaetes in the red triangle. The changes in the microbiome influence, and are influenced by, the immune state indicated as tolerization/suppression (Tol^n^) in the green boxes and activation (red boxes) at the top of the figure. This bi-directional relationship between immune activation status and microbiome status is indicated by green or red arrows to the sides of the figure; such an inter-relationship is linked to the stages of immune progression resulting in episodes of relapse and remission—inflammation/anti-pathogen response vs. anti-inflammatory/suppression of anti-pathogen response (cellular/molecular mechanisms indicated in central panel of gingival/periodontal tissues). The mechanisms associated with PD progression are indicated numerically and are as follows: **1.** Homeostatic/healthy tissue is associated with the oral microbiome, M2 anti-inflammatory Mϕs, DC/APC interaction with and immune tol^n^ by Tregs. **2.** Early act^n^ by plaque build-up and microbial changes result in M1 Mϕ and Nϕ (PMN) infiltration and act^n^, associated with gingivitis. **3.** Early ET may suppress M1 pro-inflammatory act^n^, favouring microbial replication, hence dysbiosis towards *P. gingivalis* and red complex bacteria. **4.** Changes to oral microbiome favour further M1 act^n^ and pro-inflammatory cytokine production, driving tissue-destructive, pathogen-clearing Th1-dependent CMI. **5.** In an attempt to control this CMI, tol^n^ may be initiated by PG-LPS, resulting in Treg recruitment, whereas M1 Mϕs are refractory to ET and may exhibit plasticity: changing to an M2 subset. **6.** FoxP3^+^ IL-17^+^ Tregs polarize towards Th17 cells, where IL-17 prod^n^ perpetuates inflammation by the act^n^ of Nϕs and Mϕs. **7.** Th17-driven pathology may persist, but Mϕ responses to PL-LPS result in M1 to M2 polarization, driving both tolerogenic mechanisms and immune activation by Th2-mediated B cell humoral responses. **8.** Immune cells and the cycling of act^n^/Tol^n^ by the inflammatory process and progression to PD further alter the oral microbiome (**8a**) and immune inflammatory mediators (antibodies, cytokines, ROS and MMPs) (**8b**).

The immunopathological mechanisms underlying the chronic destructive nature of PD are a complex network of dysregulated innate and adaptive immune responses in an attempt to clear pathological bacteria such as *P. gingivalis* that present as keystone pathogens resulting from inflammation and its reciprocal relationship to the dysbiosis of the oral microbiome (Figure 1). PD starts with an uncontrolled innate response mediated by Nϕs and Mϕs that result in a pro-inflammatory cytokine profile unregulatable by anti-inflammatory cytokines. These cytokine profiles and cycling between activatory and suppressive environments effectively explain the bouts of relapse and remission in the inflammatory response. During episodes of remission, ET is likely involved, which may be of benefit to the host by limiting destructive inflammatory mechanisms, but at the same time, it may be of benefit to the pathogen to allow it to recoup its numbers. This poses the question, who is ET good for? Host or pathogen? In such locations where the cytokine profile may be under continuous change depending on the local tissue environment, *P. gingivalis* virulence factors contribute to the profiles, which may play an important role in subverting a potentially pathogen-clearing Th1-mediated response to that of a Th2 humoral response that facilitates pathogen persistence. Couple this with the observations that virulence factors affect cytokine profiles; then changes in these molecules go some way to explain the emergence of Th17-mediated Nϕ activation and alveolar bone-destructive mechanisms at the expense of Treg-mediated immune tolerance and the suppression of destructive inflammation. As a consequence, PD and its inflammatory network are driven by a complexity of host factors from both innate and adaptive immunity, which is governed by a cycle of host inflammatory molecules, which in turn control the dysbiosis and predominance of pathogens such as *P. gingivalis* and their virulence factors, as well as cycling back to modulate host immunity to the advantage of the pathobiont.

*P. gingivalis* employs a diverse and highly coordinated collection of immune evasion strategies that target multiple branches of host defence; however, its influence remains elusive. Continued research is therefore essential to fully understand these pathways and identify potential therapeutic strategies to target immune evasion strategies employed by *P. gingivalis*, hence bacterial persistence and immune subversion resulting in a chronic inflammatory state. Gingipains manipulate complement activation, the phagocytic killing of infected cells, Nϕ activation and elastase/IL-8 production and cytokine skewing towards Th17, rather than Treg; the inhibition of such gingipain-mediated responses would appear to present as a valid treatment regimen [131,132,153]. Additionally, the antagonism of fimbria-induced responses, such as blocking CR3, may represent an appropriate approach to reconstituting IL-12 production, hence Th1-cell mediated responses for appropriate pathogen killing and clearance [25]. The understanding of the dynamics between pathogen expression and host responsiveness to PG-LPS is likely to indicate further therapeutic routes for the treatment of PD, but what is required? PG-LPS modification and the stimulation of TLR4-mediated inflammatory responses are required, leading to clearance mechanisms dependent on cell-mediated immunity or the induction of AMPs such as LL-37 and β-defensins [154]. If the balance is incorrect and tipped in favour of clearance activatory mechanisms, this may result in an over-zealous pro-inflammatory effect that is destructive to host tissue and may alter pathobiont dominance, hence leading to a deviation in the immune response required for microbial clearance. Conversely, if PG-LPS response potency is weak or indeed it induces a predominant ET response, this may temper tissue-destructive host inflammation but also favour bacterial growth and persistence. Thus, the manipulation of ET may only be used at the appropriate stage in PD progression or the relapse–remission cycle. Controversially, it may be considered that ET may not be involved in this pathology and that episodes of quiescence may alternatively represent periods of Mϕ polarization plasticity, effectively converting pro-inflammatory cells to anti-inflammatory cells and *visa versa*, with downstream implications for cytokine profiles. These cytokines are sensitive to degradation by *P. gingivalis*-containing biofilms [155] with regard to gingipain production and other proteinases; as such, the future therapeutic targeting of cytokine production and functionality will have to consider the expression and activity of these enzymes—possibly rationalizing a combination therapeutic approach. Finally, OMVs contain a wealth of *P. gingivalis*-associated PAMPs and virulence factors, exhibiting both immune activatory and suppressive effects; such OMVs have been suggested as vaccine components to resist the accumulation of this keystone pathogen [156]. Overall, the therapeutic treatment of PD can only be achieved by obtaining a comprehensive understanding of the immune–microbiome axis: the complete understanding of the bacterial activation of innate immunity, the cytokine profiles elicited, the early effects of dysbiosis and the cycling between bouts of inflammatory activation and tolerization allow for an appreciation for PD progression, utilizing adaptive immune mechanisms. These adaptive responses, which change from Th1-driven CMI to Treg/Th17 plasticity, result in further tissue destruction and, finally, Th2-B-cell humoral responses. As such, the immunopathological mechanisms driving PD and the pathogen-derived evasion strategies modulating host defences are highly variable and dependent on the stage of progression. The appreciation of these specific host–pathogen mechanisms at distinct stages may clarify novel therapeutic approaches in the treatment of PD.

## Figures and Tables

**Table 1:** Cytokine profiles in periodontal disease.

Cytokine	Sample	Role in PD/Notes	PD Ref
**TNFα**	GCF, serum, perio tissue, PD-Mϕs	Bone resorp^n^, synergy with IL-1. Acute phase, prevents repair.	[57,58,59]
**IL-1α**	GCF, perio tissue, PD-Mϕs, gingival fibroblasts	Alarmin. Protease synthesis.	[58,60,61]
**IL-1β**	GCF, perio tissue, PD-Mϕs, saliva, serum	IL-1β^+^ Mϕs elevated in PD. MMP prod^n^. Bone resorp^n^.	[58,62,63,64,65]
**IL-1Ra**	GCF, perio tissue	Up- and down-regulated in PD. Antagonize pro-inflammatory effect of IL-1. Limit bone resorp^n^.	[66,67,68]
**IL-4**	GCF	PD—low, elevated in remission. M2 and Th2 polarization.	[59,66,69,70]
**IL-6**	GCF, perio tissue and plasma, PD-Mϕs	PD—elevated. B cell act^n^ and Ig prod^n^. Periodontal damage and bone loss. OC Diff^n^.	[58,69,71,72]
**IL-8**	GCF, perio tissue, PD-Mϕs	Neutrophil migration to gingival sulcus. NET formation.	[58,73,74,75]
**IL-10**	Perio tissue	CD8^+^ T prod^n^ of IL-10. Anti-inflamm. M2/Treg polarization. Induces TIMPs, inhibits MMPs. Sometimes supp^d^.	[64,76,77]
**IL-11**	GCF, perio tissue	Decreased IL-11. Anti-inflamm.	[78,79,80]
**IL-12**	Perio tissue	PD: IL-12^+^ B cells elevated. Th1 diff^n^ NK act^n^. Synergizes with IL-18.	[64,76,81]
**IL-13**	PD perio tissue	Induct^n^ of periostin and Th2-mediated tissue destruction in periodontium.	[77,82]
**IL-15**	Perio tissue	IL15: decr. In PD. Incr. iNOS/NO in gingival epithelial cells. Synergizes with RANKL in osteoclastogenesis.	[69,77,83]
**IL17**	GCF, sera, perio tissue	Elevated in PD. NK act^n^. Mϕ act^n^. Nϕ act^n^. IL-17 prod^n^ by Th17 and Nϕs.	[78,84,85,86,87,88]
**IL-18**	GCF, saliva, serum	PD: NK act^n^, Nϕ act^n^ and Th1: IFNγ prod^n^. Synergy with IL-12. IL-18 act^n^ by NLRP3.	[65,89,90,91,92]
**IL-21**	Saliva	Stage III Grade C periodontitis: pro-inflamm, released by Th17 cells, supp^n^ Th2-IL-13. IL-21 elevated in saliva.	[93,94,95]
**IL-23**	Perio tissue, GCF, saliva, PD Mϕs	Th17 diff^n^.	[96,97,98,99]
**IL-27**	Perio tissue, GCF, saliva	IL-27 decreased—anti-inflammatory: supp^n^ of IL-17.	[88,99,100]
**IL-33**	Perio tissue, GCF	PD:Alarmin, ILC2, Treg, Th2 CK and NK Act^n^, osteoclastogenesis and RANKL—alveolar bone loss, microbial dysbiosis.	[101,102,103]
**IL-35**	GCF, perio tissue	PD/CP. Immune tolerance. Diff^n^ and functionality of iTreg35 cells.	[88,104]
**IL-36**	PD perio tissue, GCF, saliva	DC, Mϕ and Nϕ chemotaxis, amplif^n^ of IL-17 secretion. Stim^n^ Th17 chemokines and bone resorp^n^.	[105,106,107,108]
**IL-36Ra**	PD perio tissue	Anti-inflamm. Antagonistic to IL-36 functionality. Down-regulated expression.	[105]
**IL-37**	Perio tissue	PD: anti-inflamm. Smad-3 binding: TGFβ activity. Suppresses innate and adaptive IRs. Inhibitor of IL-18.	[109,110]
**IL-38**	PD GCF, saliva	Blocks pro-inflammatory cytokines. Down-regulated expression in PD.	[107,108]
**TGFβ**	Perio tissue, GCF, saliva, serum	Anti-inflamm. Inhibits MMP synthesis. Stim^n^ of GF repair activity. Sometimes suppressed in PD.	[111,112]
**IFNγ**	Perio tissue, GCF, saliva	M1 polarization, DTH response, Th2 suppression. NK function. Induct^n^ of MHC and adhesion molecules.	[77,113,114,115]
**MCP-1** **IP-10** **VEGF**	PD: GCF	Monocyte and T-cell chemotaxis. Induct^n^ of angiogenesis; pro-inflamm.	[83,116,117,118]

Cytokine expression and/or protein production associated with PD, aggressive PD (APD) and chronic periodontitis (CP). Cytokines indicated were detected as mRNA transcripts or proteins produced and measured by immunohistochemistry staining in periodontal tissue (perio tissue) or secreted protein measured by ELISA from periodontal disease macrophages (PD-Mϕs) or in serum, saliva or gingival crevicular fluid (GCF). In addition, functional roles played by these cytokines in PD immunopathology are indicated. Cytokines indicated: IL—interleukin; TNFα—tumour necrosis factor-alpha; TGFβ—transforming growth factor-beta; IL-1/36Ra—IL-1 and IL-36 receptor antagonist; IFNγ—interferon-gamma. Abbreviations: resorp^n^, resorption; prod^n^, production; act^n^, activation; diff^n^, differentiation; inflamm. inflammatory; supp^d^, suppressed; supp^n^, suppression; induct^n^, induction; stim^n^, stimulation; amplif^n^, amplification; incr., increase; decr., decrease; NET, neutrophil extracellular trap; MMP, matrix metalloproteinase; TIMP, tissue inhibitor of metalloproteinases; NK, natural killer cell; GFs, gingival fibroblasts; IR, immune response. Cytokines labelled in green denote anti-inflammatory/regulatory cytokines. Cytokines indicated in black are pro-inflammatory and activatory.

## Data Availability

No new data were created or analyzed in this study. Data sharing is not applicable to this article.
